# Similar Efficacies of Selection Shape Mitochondrial and Nuclear Genes in Both *Drosophila melanogaster* and *Homo sapiens*

**DOI:** 10.1534/g3.114.016493

**Published:** 2015-08-21

**Authors:** Brandon S. Cooper, Chad R. Burrus, Chao Ji, Matthew W. Hahn, Kristi L. Montooth

**Affiliations:** * Department of Biology, Indiana University, Bloomington, Indiana 47405; † School of Informatics and Computing, Indiana University, Bloomington, Indiana 47405

**Keywords:** cytoplasmic sweep, mtDNA, neutrality index, tests of selection

## Abstract

Deleterious mutations contribute to polymorphism even when selection effectively prevents their fixation. The efficacy of selection in removing deleterious mitochondrial mutations from populations depends on the effective population size (*N_e_*) of the mitochondrial DNA and the degree to which a lack of recombination magnifies the effects of linked selection. Using complete mitochondrial genomes from *Drosophila melanogaster* and nuclear data available from the same samples, we reexamine the hypothesis that nonrecombining animal mitochondrial DNA harbor an excess of deleterious polymorphisms relative to the nuclear genome. We find no evidence of recombination in the mitochondrial genome, and the much-reduced level of mitochondrial synonymous polymorphism relative to nuclear genes is consistent with a reduction in *N_e_*. Nevertheless, we find that the neutrality index, a measure of the excess of nonsynonymous polymorphism relative to the neutral expectation, is only weakly significantly different between mitochondrial and nuclear loci. This difference is likely the result of the larger proportion of beneficial mutations in X-linked relative to autosomal loci, and we find little to no difference between mitochondrial and autosomal neutrality indices. Reanalysis of published data from *Homo sapiens* reveals a similar lack of a difference between the two genomes, although previous studies have suggested a strong difference in both species. Thus, despite a smaller *N_e_*, mitochondrial loci of both flies and humans appear to experience similar efficacies of purifying selection as do loci in the recombining nuclear genome.

The effective size of a population (*N_e_*) impacts how effectively selection removes deleterious mutations and fixes advantageous mutations. The unique genetics of the mitochondrial genome (mitochondrial DNA; mtDNA) are thought to reduce its *N_e_* relative to the nuclear genome, via haploid, uniparental inheritance, the mitochondrial bottleneck in the maternal germline, and a lack of recombination that decreases *N_e_* via selection on linked sites (Hill and Robertson 1966; Maynard Smith and Haigh 1974; Gillespie 2000; Meiklejohn *et al.* 2007; White *et al.* 2008; Charlesworth 2012). In addition, cytoplasmic transmission can link the mtDNA to selfish cytoplasmic elements (*e.g.*, *Wolbachia* in insects) that may sweep through populations, further decreasing mitochondrial *N_e_* and possibly increasing mitochondrial substitution rates via the fixation of slightly deleterious mutations (Shoemaker *et al.* 2004). For these reasons it has been widely hypothesized that selection is less effective in mitochondrial genomes than in their nuclear counterparts and that mitochondrial genomes may accumulate greater numbers of deleterious substitutions (Lynch 1996, 1997). Analyses of sequence data in *Drosophila* and mammals have largely supported the conclusion that mtDNA harbors significant levels of slightly deleterious polymorphism (Ballard and Kreitman 1994; Rand and Kann 1996; Nachman 1998; Rand and Kann 1998; Weinreich and Rand 2000).

*N_e_* is not the only evolutionary parameter that distinguishes mitochondrial and nuclear genomes. The distinct functional landscape of the mitochondrial genome likely affects the distribution of selective effects (*s*) of mutations that arise in this genome. Animal mitochondrial genomes typically encode regulatory information for replication and transcription nested within a hypervariable region (also known as the D-loop, control, or A+T-rich region), 22 transfer RNAs (tRNAs), two ribosomal components, and 13 protein-coding genes—all core components of oxidative phosphorylation (OXPHOS). Outside of the hypervariable region, there is little noncoding DNA in animal mtDNAs. In Drosophilids, 99% of the genome outside of the hypervariable region encodes DNA and RNA genes with highly conserved sequences that function in mitochondrial protein synthesis and aerobic respiration (Clary and Wolstenholme 1985; Wolstenholme and Clary 1985; Ballard 2000; Montooth *et al.* 2009), which suggests that the distribution of selective effects in the mtDNA may be shifted toward larger negative effects on fitness.

The mutational landscape of the mtDNA also differs from the nuclear genome. In most animal taxa, the mitochondrial mutation rate greatly exceeds that of the nuclear genome (Lynch *et al.* 2008), and the mitochondrial mutational process is also highly biased (Montooth and Rand 2008). For example, nearly all mitochondrial mutations in *D. melanogaster* change a G:C base pair to an A:T (Haag-Liautard *et al.* 2008). When combined with the strong A+T-bias in this mitochondrial genome, where 95% of third codon positions are an A or a T (Montooth *et al.* 2009), this indicates that the most commonly occurring mutations in protein-coding loci of the *Drosophila* mtDNA will change an amino acid. Relative to the nuclear genome, animal mitochondrial genomes thus experience a greater mutational pressure that can also be biased in some taxa toward nonsynonymous mutations; these are likely to have deleterious effects in a molecule that encodes such highly conserved functions.

Some of the strongest population genetic patterns in support of distinct selective pressures acting on mitochondrial and nuclear genomes come from analyses of the neutrality index (*NI*) (Rand and Kann 1996; Nachman 1998; Rand and Kann 1998; Weinreich and Rand 2000). *NI* is a summary statistic of the deviation from the neutral expectation in the McDonald-Kreitman (MK) test (McDonald and Kreitman 1991; Rand and Kann 1996) and is calculated from counts of synonymous and nonsynonymous polymorphic and divergent sites within and between related species. Weakly deleterious nonsynonymous mutations that segregate in the population, but that will not contribute to divergence, lead to a value of *NI* greater than 1. When the efficacy of selection is decreased, the expectation is that the number of segregating weakly deleterious polymorphisms will increase; this is the pattern that has been observed in mtDNA. Meta-analyses of MK tables and their associated *NI* values for mitochondrial and nuclear loci in animals have concluded that *NI* is predominantly greater than the neutral expectation of 1 for the mtDNA (Rand and Kann 1996; Nachman 1998; Rand and Kann 1998; Betancourt *et al.* 2012) and exceeds the average *NI* of the nuclear genome (Weinreich and Rand 2000). Although the relative sparseness of the data was recognized early on (Nachman 1998), and the conclusions were largely limited to how selection shapes animal mtDNA, these patterns are often taken as evidence that selection is largely ineffective in the mtDNA because of its reduced *N_e_* and that mitochondrial genomes are expected to harbor more deleterious polymorphisms than do their nuclear counterparts.

Here we revisit this pattern using new, complete mitochondrial genomes from *D. melanogaster* that we compare with published nuclear data from the same samples (Langley *et al.* 2012) and with available human data from both mitochondrial and nuclear genomes (Bustamante *et al.* 2005; Just *et al.* 2008; Rubino *et al.* 2012). We find little evidence that the effects of purifying selection differ on average between mitochondrial and nuclear genomes within flies or within humans, despite evidence that there is much reduced *N_e_* due to a lack of recombination and linkage with the cytoplasm. We discuss reasons why *NI* is, on average, similar between mitochondrial and nuclear loci, despite the distinct population genetic properties of these two genomes.

## Materials and Methods

### *D. melanogaster* mtDNA assembly, annotation, and estimates of sequence diversity

Raw sequence read files from 38 genetic lines of *D. melanogaster* from Raleigh, North Carolina (Mackay *et al.* 2012), sequenced by the 50 Genomes subproject of the *Drosophila* Population Genomics Project (Langley *et al.* 2012) were downloaded from the National Center for Biotechnology Information Sequence Read Archive. We used the Burrows-Wheeler Aligner, and specifically the fast and accurate short read alignment with Burrows-Wheeler Transform (Li and Durbin 2009), to map sequence reads to the *D. melanogaster* mitochondrial reference genome (NC_001709). We allowed up to five gaps, five gap extensions, and five mismatches per aligned read, but few reads needed such flexibility and most were filtered out in later steps. Using SAMtools, we postprocessed the alignments to filter out low-quality alignments and to detect single-nucleotide polymorphisms (SNPs) (Li *et al.* 2009). SNPs with a quality score greater than 20 and indels with a quality score greater than 50 were kept for further analyses. We then generated a consensus sequence for each of the *D. melanogaster* mtDNAs listed in Supporting Information, Table S1. Because of the high variance in coverage across the hypervariable region, we did not include this region in our final assemblies or analyses.

We annotated the consensus sequence for each mtDNA using the GenBank annotation of the *D. melanogaster* reference sequence (NC_001709). Using ClustalW (Larkin *et al.* 2007), we performed a whole-genome alignment, as well as gene-specific alignments, of each consensus sequence to the reference sequence and to the outgroup species *Drosophila yakuba* (NC_001322). There are very few indels in the protein-coding regions of *Drosophila* mtDNA (Montooth *et al.* 2009), making alignment straightforward. From these alignments we calculated expected heterozygosity (*π*), the number of segregating sites (*S*), and Watterson’s *θ_W_* (Watterson 1975) as measures of sequence diversity. The mitochondrial haplotype network was inferred from 80 segregating sites in the coding region of the mtDNA for which there were no missing or ambiguous data using TCS version 1.21 (Clement *et al.* 2000).

### Tests for recombination in the *D. melanogaster* mtDNA

We estimated linkage disequilibrium (LD) between all pairs of mitochondrial SNPs using the statistic *D′* (Lewontin 1964), where *D′* = 0 indicates no LD and |*D*′| = 1 indicates perfect LD. Because recombination erodes LD as a function of distance, a negative correlation between |*D*′| and genetic distance between pairs of SNPs has been used as evidence for recombination in mtDNA (Awadalla *et al.* 1999). To test this prediction, we looked for significant negative correlations between |*D*′| and genetic distance. We also conducted these same tests using another statistical measure of association, *r^2^* (Hill and Robertson 1966), which is more robust to variation in mutation rates (Awadalla *et al.* 1999; Meunier and Eyre-Walker 2001; Innan and Nordborg 2002). We calculated these correlations by using a variety of minor allele frequency cutoffs. We also tested for the presence of all four genotypes at pairs of SNPs (the “four-gamete test”; Hudson and Kaplan 1985) using DNAsp version 5 (Rozas *et al.* 2003).

### Neutrality tests

Using *π* and *θ_W_*, we calculated Tajima’s *D* (Tajima 1989), which is expected to be 0 for a neutrally evolving locus. Demographic effects will skew the site-frequency spectrum of both synonymous and nonsynonymous polymorphisms at a locus. Contrasting Tajima’s *D* between nonsynonymous and synonymous polymorphisms therefore tests whether nonsynonymous alleles experience a greater skew in frequency relative to putatively neutral synonymous alleles, indicative of selection (Rand and Kann 1996). We implemented this analysis using the heterogeneity test (Hahn *et al.* 2002), which simulates 10,000 genealogies with no recombination by using the values of synonymous and nonsynonymous *S* calculated from the data and compares the estimated difference in Tajima’s *D* to the random distribution of differences between synonymous and nonsynonymous polymorphisms. We calculated several other summaries of the site-frequency spectrum, including Fu and Li’s *D*, which characterizes the proportion of mutations on external and internal branches of a genealogy (Fu and Li 1993) and Fay and Wu’s *H*, which tests for an excess of high-frequency, derived alleles in a sample relative to the neutral expectation (Fay and Wu 2000). These latter statistics were calculated using a set of 80 segregating sites in the coding region of the mtDNA for which there were no missing or ambiguous data. Significance was determined using 10,000 coalescent simulations as implemented in DNAsp version 5 (Rozas *et al.* 2003).

We constructed MK (McDonald and Kreitman 1991) two-by-two contingency tables of counts of nonsynonymous and synonymous polymorphisms (*P_N_* and *P_S_*) within *D. melanogaster* and nonsynonymous and synonymous fixed differences (*D_N_* and *D_S_*) between *D. melanogaster* and either *D. yakuba* or *Drosophila simulans*. Polymorphic sites within *D. melanogaster* only contributed to fixed differences if the allele in the outgroup sequence was not present in *D. melanogaster*. We tested for significant deviations from neutrality by using the Fisher’s exact tests of the MK table in R version 2.15.1 (R Core Team 2012). We calculated *NI*—the ratio of *P_N_/P_S_* to *D_N_/D_S_*—as a summary statistic of the MK table (Rand and Kann 1996). Assuming that selection is constant, the neutral expectation is that *D_N_/D_s_* will equal *P_N_/P_S_* (Kimura 1983; McDonald and Kreitman 1991), and *NI* is expected to be 1. When calculating *NI* for any gene with a count of 0 in any cell of the MK table, we added a count of 1 to all cells (Sheldahl *et al.* 2003; Presgraves 2005). Twenty-three percent of 13 *D. melanogaster* mitochondrial genes, 9.5% of 6113 *D. melanogaster* nuclear genes, 0% of 13 *H. sapiens* mitochondrial genes, and 73% of 11,624 *H. sapiens* nuclear genes required these additional counts. If the MK test is significant, an *NI* value less than 1 indicates a significant excess of nonsynonymous fixed differences, whereas an *NI* value greater than 1 indicates a significant excess of nonsynonymous polymorphisms. We also calculated $Z^{*}=Log_{10}\left(\frac{\left(D_{N}+1\right)\left(P_{S}+1\right)}{\left(D_{S}+1\right)\left(P_{N}+1\right)}\right)$, as in Presgraves (2005), the sign of which is more intuitive; negative values are consistent with an excess of weakly deleterious (negatively selected) polymorphisms and positive values are consistent with an excess of advantageous (positively selected) substitutions.

The short length and low *D_N_* values for mitochondrial genes upwardly biases *NI* (Weinreich and Rand 2000), and we initially used *D. yakuba* as the outgroup to increase the amount of divergence. However, using *D. yakuba* to calculate divergence also increases the potential for multiple substitutions at silent sites. Because of this, we constructed MK tables for the 13 mitochondrial protein-coding genes using a range of taxa and methods that capture different amounts of sequence divergence (Table S2 and Table S3). We used both *D. simulans* and *D. yakuba* to polarize changes on the branch leading to *D. melanogaster*, which resulted in very few nonsynonymous substitutions and highly variable *NI* values (Table S2). We also used either *D. simulans* or *D. yakuba* singly to calculate divergence with *D. melanogaster* in two ways. The “more-inclusive” approach included codons that were missing data in some lines, and averaged across all possible mutational pathways between codons with multiple substitutions to estimate *D_N_* and *D_S_* (Nei and Gojobori 1986). The “less-inclusive” method omitted codons with any missing data, omitted mtDNA SRX022291 (which contained more missing data than any other mtDNA) and calculated divergence using the mutational path that minimized *D_N_* between codons with multiple substitutions. Unless otherwise noted, the more-inclusive method using *D. yakuba* as outgroup is presented and discussed.

In addition to using counts for single genes, we also analyzed MK tables of the summed counts of polymorphic and divergent sites for each of the mitochondrial-encoded OXPHOS complexes: Complex I (NADH dehydrogenase, *ND*), Complex IV (cytochrome *c* oxidase), and Complex V (ATP synthase). *Cytochrome B* is the only Complex III gene encoded by the mtDNA. Stoletzki and Eyre-Walker (2011) emphasize that contingency data generally should not be summed, particularly when there is heterogeneity among contingency tables, and they provide an unbiased estimator of overall *NI* for combining counts across genes, $NI_{TG}=\frac{\sum^{\text{​}}D_{Si}P_{Ni}/\left(P_{Si}+D_{Si}\right)}{\sum^{\text{​}}P_{Si}D_{Ni}/\left(P_{Si}+D_{Si}\right)}$. We calculated this statistic and used the DoFE software package (Stoletzki and Eyre-Walker 2011) to calculate bootstrap confidence intervals and to conduct Woolf’s tests of homogeneity (Woolf 1955). The only data set with significant heterogeneity was the *D. melanogaster* nuclear gene set (*P <* 0.0001). Similar statistics were used to analyze polymorphism and divergence in the human data sets, as well as in a subset of the mitochondrial haplotypes reported in our study that were independently sequenced and assembled by Richardson *et al.* (2012) (Table S4).

### Comparisons of mitochondrial and nuclear *NI* in flies and humans

To compare patterns of polymorphism and divergence between mitochondrial and nuclear genomes, we obtained existing data for nuclear genes in *D. melanogaster* and for nuclear and mitochondrial genes in *Homo sapiens*. Counts of polymorphism and divergence for *D. melanogaster* nuclear genes were obtained from the *Drosophila* Population Genomics Project analysis of the same 38 genomes from Raleigh, North Carolina, with divergence polarized along the *D. melanogaster* lineage and using the mutational path that minimized *D_N_* between codons with multiple substitutions (Langley *et al.* 2012). The human nuclear data from Bustamante *et al.* (2005) included counts of polymorphic and divergent sites from 19 African Americans and 20 European Americans, using the chimpanzee *Pan troglodytes* as an outgroup. We calculated the number of polymorphic and divergent sites for human mitochondrial genes by using mtDNA sequences from 19 African Americans (Just *et al.* 2008), 20 European Americans (Rubino *et al.* 2012), and the chimpanzee mitochondrial reference genome D38113.1 (Horai *et al.* 1995) using the “more-inclusive” method described previously. We also analyzed subsets of the human mitochondrial data to illustrate the sensitivity of *NI* to sampling (Table S5 and Table S6). Comparisons of the distributions of *NI* and Z* between gene sets were performed using Mann−Whitney *U*-tests in R version 2.15.1 (R Core Team 2012).

### Data availability

File S1 contains the 38 D. *melanogaster* assembled mtDNA genomes used in this study aligned with *D. yakuba* (NC_001322). Human mtDNA sequence data are available in GenBank, and the accession numbers are listed in Table S6.

## Results

### An excess of low- and high-frequency, derived mitochondrial polymorphisms

**Figure 1:**
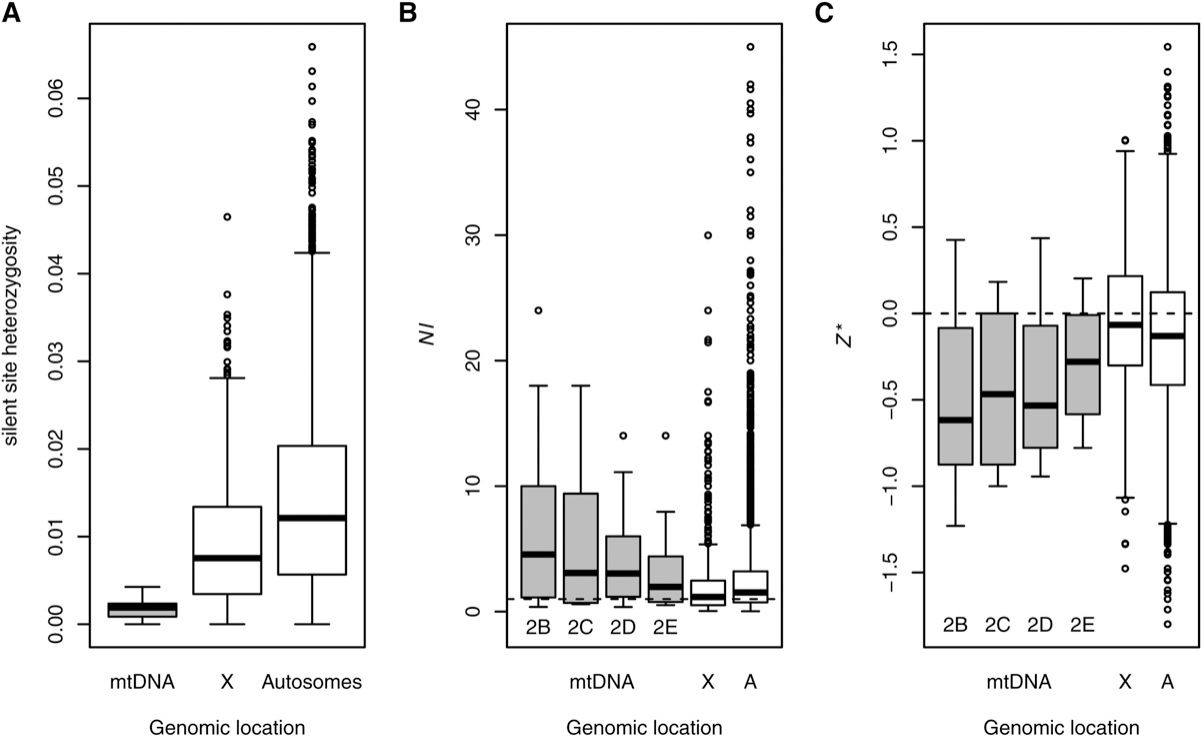
Effect of genomic location on silent-site heterozygosity and on summary statistics of polymorphism and divergence in *D. melanogaster*. (A) Genomic location has a significant effect on per-site silent-site heterozygosity (*P_MWU_* < 0.001 for all pairwise contrasts), consistent with predicted differences in the effective population size (*N_e_*). The ratio of median mitochondrial to autosomal silent site heterozygosity was 0.157, less than predicted for neutral sites if mitochondrial *N_e_* is one quarter that of the autosomes. mitochondrial DNA (mtDNA), X-chromosome, and autosome data sets contained 12, 1255, and 8073 genes, respectively. (B, C) Distributions of neutrality index (*NI*) and Z* are similar between mitochondrial and autosomal (abbreviated as A) genes, with moderately significant differences between mitochondrial and X-linked (abbreviated as X) genes. The four mtDNA boxes represent estimates from the corresponding MK tables in Table S2, B−E that used either *D. simulans* (Table S2, B−C) or *D. yakuba* (Table S2, D−E) as the outgroup. Dashed lines represent the neutral expectations for these statistics. Three nuclear loci for which *NI* exceeded 50 were excluded from (B) to improve visualization. Statistical results are presented in Table S2. mtDNA, X-chromosome, and autosome data sets contained 13, 712, and 5401 genes, respectively.

We assembled 14,916 bp of sequence containing the transcribed regions of the mtDNA with a median coverage of 32x for 38 genetic lines sampled from a single population of *D. melanogaster* in Raleigh, North Carolina (Langley *et al.* 2012; Mackay *et al.* 2012) (Table S1). More than 98% of these nucleotides encode the 13 protein-coding genes, 22 tRNAs, and two ribosomal RNAs. The per-site expected heterozygosity in this region (*π*) of the mtDNA was 0.0008. We identified 137 segregating sites in this population sample, 103 of which were in protein-coding genes. Median heterozygosity in protein-coding genes was 0.0023 per synonymous site and 0.0002 per nonsynonymous site. Silent site heterozygosity was significantly lower in mitochondrial genes relative to nuclear genes (Mann−Whitney *U*, mtDNA *vs.* X chromosome, *P_MWU_* = 0.00002; mtDNA *vs.* autosomes, *P_MWU_* < 0.00001) and was only 0.16 times that of the autosomes (Figure 1A), lower than what is expected if the mtDNA has an effective population size that is one-quarter that of the autosomes.

**Figure 2:**
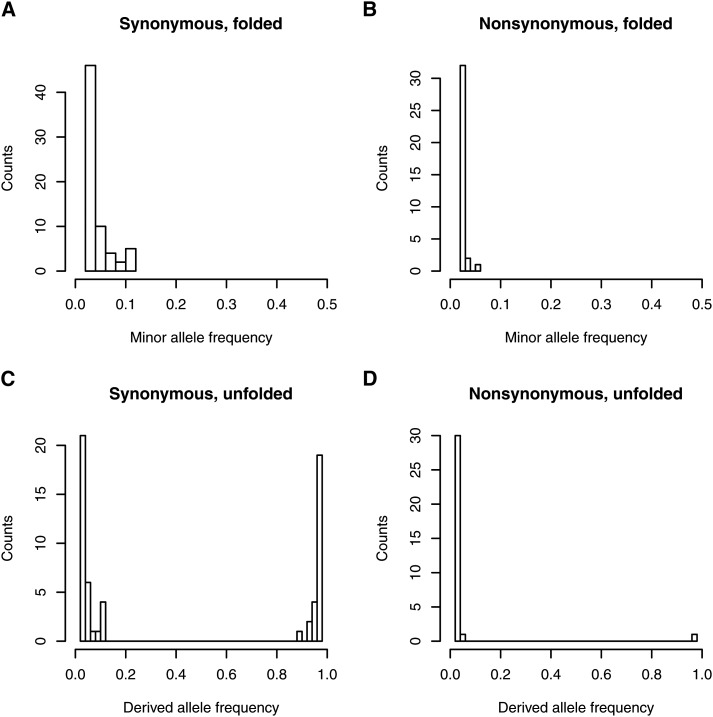
Site-frequency spectra of synonymous and nonsynonymous polymorphisms in the *D. melanogaster* mitochondrial DNA. (A, B) Folded site-frequency spectra for synonymous and nonsynonymous segregating sites across the mitochondrial protein-coding region reveal that mitochondrial polymorphisms are skewed to low frequencies. (C, D) Unfolded site-frequency spectra reveal that derived, synonymous polymorphisms are almost equally likely to be at low frequency (56% of 59 sites at frequencies less than 0.11) or nearly fixed (44% of 59 sites at frequencies greater than 0.89), while derived, nonsynonymous polymorphisms are nearly always present as singletons (94% of 32 sites). There are essentially no mitochondrial polymorphisms at intermediate frequencies. Sites were omitted from the unfolded site frequency spectra if neither allelic state was shared with *D. yakuba*. The number of sites included in each distribution is 67 (A), 35 (B), 59 (C), and 32 (D).

In addition to the scarcity of segregating sites in the *D. melanogaster* mtDNA, polymorphisms at these sites were skewed toward low frequencies (Figure 2, A and B), as evidenced by consistently negative values of Tajima’s *D* (Table 1). Tajima’s *D* across the mtDNA was −2.607 and differed significantly from the neutral expectation of 0 (*S* = 80 for coding sites with no missing data, *P <* 0.0001), as did Fu and Li’s *D* (*D* = −2.67, *P <* 0.05). The minor allele frequency for unpolarized synonymous polymorphisms was always less than 11%, and all but one of the nonsynonymous polymorphisms were singletons (Figure 2, A and B). Using *D. yakuba* as an outgroup revealed that the derived allele was nearly fixed for 44% of segregating synonymous sites, whereas there was only a single derived nonsynonymous polymorphism at high frequency (Figure 2, C and D). Using both *D. simulans* and *D. yakuba* to polarize mutations did not qualitatively change this result (Figure S1). Thus, the mitochondrial genome was essentially devoid of intermediate-frequency polymorphisms, with derived synonymous mutations at either very high (greater than 89%) or very low (less than 11%) frequencies and nearly all derived nonsynonymous polymorphisms at frequencies less than 5%. This skew toward high-frequency, derived alleles resulted in a significant negative value of Fay and Wu’s *H* statistic (*H* = −41.2, *P* = 0.005).

**Table 1:** Synonymous and nonsynonymous variation in *D. melanogaster* mitochondrial genes and OXPHOS complexes

Gene/Complex*^a^*	Synonymous Sites*^b^*					Nonsynonymous Sites				
	n (bp)	*S*	*π*	*Ө_W_*	*D*	n (bp)	*S*	*π*	*Ө_W_*	*D*
*COI*	347	16	1.57	3.81	−1.91	1183	0	0	0	Undef
*COII*	142	5	0.31	1.19	−1.90	539	1	0.05	0.24	−1.13
*COIII*	169.33	9	0.72	2.14	−1.96	613.67	1	0.05	0.24	−1.13
*ND1*	194	3	0.16	0.71	−1.72	739	3	0.16	0.71	−1.72
*ND2*	198	5	0.41	1.19	−1.69	822	6	0.34	1.43	−2.07
*ND3*	68	1	0.05	0.24	−1.12	280	1	0.05	0.24	−1.12
*ND4*	273.33	7	0.51	1.67	−1.95	1061.67	7	0.38	1.67	−2.17
*ND4L*	56	1	0.05	0.24	−1.13	229	2	0.11	0.48	−1.49
*ND5*	351	10	0.68	2.38	−2.16	1365	4	0.21	0.95	−1.88
*ND6*	103.33	4	0.26	0.95	−1.75	415.67	2	0.11	0.48	−1.49
Complex I	1243.66	31	2.12	7.38	−2.48	4912.34	25	1.35	5.95	−2.64
Complex III	239	5	0.55	1.19	−1.38	892	2	0.16	0.48	−1.29
Complex IV	658.33	30	2.60	7.14	−2.21	2335.67	2	0.11	0.48	−1.49
Complex V	174.67	2	0.16	0.71	−1.72	650.33	6	0.32	1.43	−2.10
Total	2315.66	68	5.43	16.42	−2.44	8790.34	35	1.93	8.33	−2.70

OXPHOS, oxidative phosphorylation; Undef, undefined; *CO*, cytochrome *c* oxidase; *ND*, NADH dehydrogenase; *ATPase*, ATP synthase.

a Complex I (*ND*), 7 loci; Complex III (*Cytochrome B*), 1 locus; Complex IV (*CO*), 3 loci; Complex V (*ATPase*), 2 overlapping loci.

b For synonymous and nonsynonymous sites, we calculated the number of segregating sites (*S*), heterozygosity (*π*), Watterson’s *Ө_W_*, and Tajima’s *D*. The heterogeneity test for differences between synonymous and nonsynonymous *D* was never significant (*P* > 0.35 for all comparisons).

### A partial sweep in the *D. melanogaster* mtDNA

**Figure 3:**
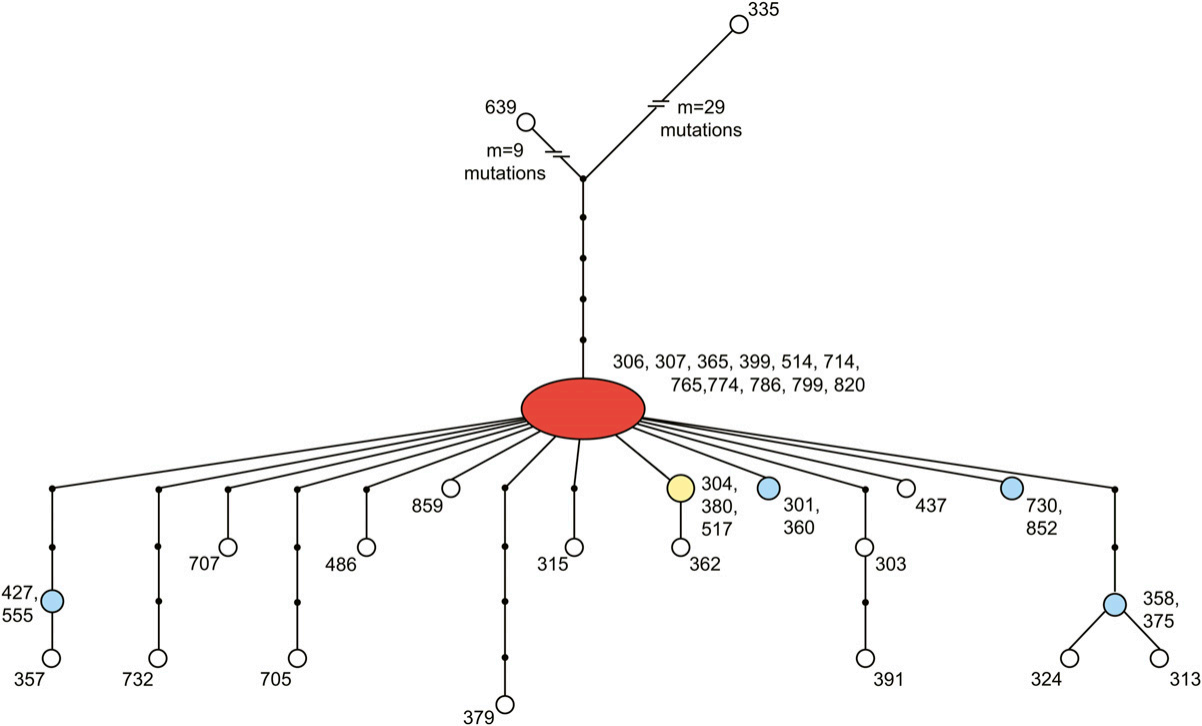
Haplotype network for 38 *D. melanogaster* mitochondrial DNAs (mtDNAs) sampled from Raleigh, North Carolina. The network, inferred from 80 coding region single-nucleotide polymorphisms (SNPs) with no missing information, reveals that nearly 30% of individuals sampled (11/38) share the same common haplotype (red) and an additional 65% of individuals carry a haplotype only a few mutations away from this haplotype. This common set of mitochondrial haplotypes is highly diverged from the two other mtDNAs sampled in the population; lines RAL-639 and RAL-335 differ from the common haplotype at 14 and 34 SNPs, respectively. At least one of these two haplotypes carries the ancestral state (shared with *D. yakuba*) at 38% of these SNPs. Numbers represent the Raleigh line carrying the haplotype. Red, yellow, blue, and white nodes were present in 11, 3, 2, and 1 lines, respectively.

The large fraction of derived alleles at high frequencies is a consequence of the haplotype structure of this sample (Figure 3). Nearly 30% of individuals in this population shared an identical mitochondrial haplotype, and an additional 66% of individuals differed from this haplotype by only one to five mutations. The two remaining haplotypes (RAL-639 and RAL-335) were highly divergent from this common haplotype group, contributing nearly half of the segregating sites to the population sample. These two haplotypes shared the ancestral state with *D. yakuba* at 17 of the 23 derived high-frequency synonymous polymorphisms (*i.e.*, they have the low-frequency ancestral allele). When these two haplotypes were removed from the analysis, there remained a strong skew toward rare alleles (Tajima’s *D* = −2.31, *P* < 0.01; Fu and Li’s *D* = −3.14, *P* < 0.02), but Fay and Wu’s *H*, which is sensitive to the number of high-frequency derived alleles, was only weakly significant (*H* = −10.34, *P* = 0.043). The remaining six derived, high-frequency synonymous polymorphisms, as well as the single derived, high-frequency nonsynonymous polymorphism, were the result of single mtDNAs within the common haplotype group having the same allelic state as *D. yakuba*. Given the lack of recombination in the mtDNA, these are likely new, rather than ancestral, mutations. Six of these seven mutations would have changed a C or G to a T or A, consistent with the mutation bias in the *D. melanogaster* mtDNA (Haag-Liautard *et al.* 2008).

### No evidence for recombination in the *D. melanogaster* mtDNA

We tested for a negative correlation between LD and the distance between each pair of polymorphic sites in the *D. melanogaster* mitochondrial genome, as a signature of the decay of LD over distance via recombination (Awadalla *et al.* 1999). There was no evidence for a decrease in LD with increasing distance between sites, regardless of the measure of LD or the minor allele cutoff used (Table S7). There were no pairs of polymorphic sites for which all four gametes were present (Hudson and Kaplan 1985; Bruen *et al.* 2006), further supporting an absence of effective recombination.

### Weakly deleterious polymorphism in the *D. melanogaster* mtDNA

The skew in the site-frequency spectrum toward rare alleles (Figure 2) resulted in negative values of Tajima’s *D* across the entire mtDNA (Table 1). However, there was no evidence that the skew toward rare alleles differed between synonymous and nonsynonymous polymorphisms (Figure 2, A and B)—heterogeneity tests (Hahn *et al.* 2002) of Tajima’s *D* between synonymous and nonsynonymous sites were never significant (*P* > 0.35 for all genes and complexes). However, unfolding the site frequency spectra revealed that the large number of high-frequency, derived sites were nearly all synonymous (Figure 2, C and D), suggesting that the haplotype that has increased in frequency carried many more synonymous than nonsynonymous polymorphisms. Given that the mutation bias in the *D. melanogaster* mtDNA greatly favors nonsynonymous mutations (Haag-Liautard *et al.* 2008), this pattern suggests a history of effective purifying selection removing mitochondrial haplotypes that contain nonsynonymous polymorphisms. Furthermore, all nonsynonymous polymorphisms that have arisen on the common mitochondrial haplotype are present at very low frequencies.

The current distribution of polymorphisms relative to divergence in *D. melanogaster* showed little evidence for a large and significant excess of segregating deleterious polymorphisms. Across MK tables, no single gene departed significantly from the neutral expectation after Bonferroni correction (*P* < 0.05/13) (Table 2 and Table S2). For the entire set of protein-coding mitochondrial genes, there was a slight excess of nonsynonymous polymorphism relative to the neutral expectation, as indicated by moderately significant MK tests [Fisher’s exact test, *P_FET_* ranged from 0.0004 to 0.041 across methods (Table S2)] and values of *NI_TG_* that ranged from 1.67 to 2.57 across methods, with confidence intervals that did not include the neutral expectation of 1 (Table S2). There was some OXPHOS-complex specificity to this result—Complexes I (*ND*) and V (*ATPase*) tended to deviate significantly from neutrality with *NI* values greater than 1, whereas Complex IV (*CO*) was consistent with the neutral expectation (Table 3 and Table S3).

**Table 2:** Counts of polymorphic (*P*) and divergent (*D*) nonsynonymous (*_N_*) and synonymous (*_S_*) sites along with summary statistics of the MK table for *D. melanogaster* mitochondrial genes

Gene	*P_N_**^a^*	*P_S_**^a^*	*D_N_**^a^*	*D_S_**^a^*	*NI**^b^*	Z*^c^	*P_FET_**^d^*
*ATPase6*	5	2	11	35	7.955	−0.778	0.021
*ATPase8*	1	0	2	8	6.000	−0.778	0.273
*COI*	0	16	8	101	0.667	0.176	0.595
*COII*	1	5	6	39	1.300	−0.280	1.000
*COIII*	1	9	8.5	47.5	0.621	−0.009	1.000
*Cyt-b*	2	5	17.5	67.5	1.543	−0.267	0.641
*ND1*	3	3	11	45	4.091	−0.584	0.122
*ND2*	6	5	25	41	1.968	−0.275	0.334
*ND3*	1	1	5	22	4.400	−0.584	0.377
*ND4*	7	7	24	63	2.625	−0.408	0.120
*ND4L*	2	1	1	7	14.00	−0.778	0.152
*ND5*	4	10	55.833	107.167	0.768	0.063	0.775
*ND6*	2	4	21.5	22.5	0.523	0.203	0.669

MK, McDonald-Kreitman; *NI*, neutrality index; *ATPase*, ATP synthase; *CO*, cytochrome *c* oxidase; *Cyt-b*, *Cytochrome B*; *ND*, NADH dehydrogenase.

a MK counts from the “more-inclusive” method. Values from other methods are in Table S2.

b A count of 1 was added to each cell when calculating $NI=\frac{D_{S}P_{N}}{D_{N}P_{S}}$ for any gene with a zero count in any cell.

c $Z^{*}=Log_{10}\left(\frac{\left(D_{N}+1\right)\left(P_{S}+1\right)}{\left(D_{S}+1\right)\left(P_{N}+1\right)}\right)$, as in Presgraves (2005).

d *P*-value from Fisher’s exact test of the MK table.

**Table 3:** Summary statistics of the MK table for mitochondrially encoded OXPHOS complexes and nuclear genes

Species	Genome	Gene Set*^a^*	*NI**^b^*	*NI_TG_**^c^*	*Z**^b^*	*P_FET_**^d^*
*D. mel*	mtDNA*^e^*	Complex I	1.73	1.59 (0.94, 3.17)	−0.238	0.070
	mtDNA	Complex IV	0.56	0.55 (0, 1.30)	0.255	0.750
	mtDNA	Complex V	9.92	9.64 (undef)	−0.997	0.007
	mtDNA	All coding	1.59 (1.97,3.57,3.89)	1.67 (1.03, 2.86)	−0.201 (-0.28,-0.33,0.36)	0.041
	Nuclear	Autosomes	1.16 (1.52,2.83,4.25)	1.39 (1.35, 1.43)	−0.064 (-0.13,-0.15,0.42)	<1e-6
	Nuclear	X chrom	0.91 (1.17,2.21,3.24)	1.02 (0.94, 1.10)	0.041 (-0.07,-0.06,0.41)	0.001
*H. sap*	mtDNA	Complex I	1.19	1.20 (0.56, 2.40)	−0.074	0.475
	mtDNA	Complex IV	1.98	2.00 (0.86, 3.61)	−0.296	0.402
	mtDNA	Complex V	1.62	1.66 (0.79, 2.42)	−0.209	0.127
	mtDNA	All coding	1.46 (1.38,1.95,1.38)	1.48 (0.90, 2.24)	−0.165 (-0.23,-0.23,0.32)	0.034
	Nuclear	All coding	1.51 (1.39,2.28,2.85)	1.57 (1.51, 1.63)	−0.180 (-0.12,-0.13,0.40)	<1e-6

MK, McDonald-Kreitman; OXPHOS, oxidative phosphorylation; *NI*, neutrality index; *D. mel*, *D. melanogaster*; mtDNA, mitochondrial DNA; undef, undefined; X chrom, X chromosomes; *H. sap*, *H. sapiens*.

a Complex I (*ND*) seven loci; Complex IV (*CO*) three loci; Complex V (*ATPase*), two overlapping loci. Complex II is nuclear encoded and Complex III has only a single mitochondrial locus, *Cyt-b*.

b *NI* and $Z=Log_{10}\left(\frac{D_{N}P_{S}}{D_{S}P_{N}}\right)$ were calculated using counts of *P_N_*, *P_S_*, *D_N_*, and *D_S_* summed across genes within gene sets. Median, mean, and SD provided for whole genome.

c $NI_{TG}=\frac{\sum^{\text{​}}D_{Si}P_{Ni}/\left(P_{Si}+D_{Si}\right)}{\sum^{\text{​}}P_{Si}D_{Ni}/\left(P_{Si}+D_{Si}\right)}$ with confidence intervals from 5000 bootstrap samples (Stoletzki and Eyre-Walker 2011).

d *P*-value from Fisher’s exact test of the MK table.

e *D. melanogaster* mtDNA data from the “more-inclusive” method. Values from other methods are in Table S3.

Analysis of 36 of the 38 mitochondrial haplotypes in our sample that were independently sequenced and assembled by Richardson *et al.* (2012) confirmed these patterns (Table S4). When counts of polymorphism and divergence differed between datasets, they typically differed by a single count. The exception was in several Complex I (*ND*) genes, for which our assembled mtDNAs had a small number of additional nonsynonymous polymorphisms relative to the Richardson *et al.* (2012) data set (*ND* genes, *df* = 6, *P_MWU_*_,_ *_paired_* = 0.021; all other genes, *df* = 5, *P_MWU_*_,_ *_paired_* = 1) that resulted in slightly greater values of *NI* (*ND* included, *df* = 12, *P_MWU_*_,_ *_paired_* = 0.016; all other genes, *df* = 5, *P_MWU_*_,_ *_paired_* = 1). This was not due to absence of two mitochondrial haplotypes in the Richardson *et al.* (2012) data set, and the additional polymorphisms in our data were not clustered on any single mitochondrial haplotype. The reduced number of nonsynonymous polymorphisms in the Richardson *et al.* (2012) data provided even less support for an excess of nonsynonymous segregating variation in the mitochondrial genome. Summed counts of polymorphism and divergence for the entire set of mitochondrial-encoded proteins in this dataset did not deviate from the neutral expectation (*P_FET_* = 0.423), and the confidence intervals on *NI_TG_* for mitochondrial-encoded proteins contained the neutral expectation of 1 (*NI_TG_* = 0.821, 95% confidence interval = 0.386 to 1.90).

### On average, *NI* is similar for mitochondrial and nuclear genes in flies

Although there is a weak signature of an excess of nonsynonymous segregating variation in the *D. melanogaster* mitochondrial genome, both mitochondrial and nuclear gene sets have median *NI*, Z*, or *NI_TG_* values that deviate in the same manner from the neutral expectation, indicative of both genomes harboring weakly deleterious polymorphisms. Furthermore, the distribution of *D. melanogaster* mitochondrial gene *NI* values was contained within that of the nuclear genes, with many nuclear genes having both more positive and more negative values of *NI* and Z* (Figure 1, B and C). Weakly significant differences between mitochondrial and nuclear gene *NI* were affected by the genomic location of nuclear genes (Table S2), as X-linked genes had significantly lower values of *NI* and more positive Z* values relative to autosomal genes (*P_MWU_* < 1e-6 for both statistics) (Figure 1, B and C). Because of this, mitochondrial gene *NI* differed significantly from X-linked genes (*P_MWU_* ranged from 0.007 to 0.065) but not from autosomal genes (*P_MWU_* ranged from 0.047 to 0.325), with similar patterns for Z* (mtDNA *vs.* X, *P_MWU_* ranged from 0.002 to 0.019; mtDNA *vs.* autosomes, *P_MWU_* ranged from 0.012 to 0.104). The lower counts of polymorphic sites in the assembled mtDNAs from Richardson *et al.* (2012) provided less support for genomic differences in MK statistics. Neither *NI* nor Z* differed significantly between the mitochondria and either the X or the autosomes (*NI*, *P_MWU_* > 0.441 for both comparisons; Z*, *P_MWU_* > 0.471 for both comparisons).

The moderate levels of significance associated with some of these contrasts, and the sensitivity of these contrasts to small differences in MK counts and methods, suggest that although there is a trend for mitochondrial genes to have larger *NI* (and more negative Z*) values relative to nuclear genes, the differences between genomes are not large. Contrasts with mitochondrial genomes may have low power due to the smaller number of genes and low levels of nonsynonymous polymorphism and divergence, relative to nuclear genomes. However, the mitochondrial data are not a sample of genes, as they represent the complete protein-coding complement of this genome. Nevertheless, a traditional power analysis suggests that we would require an 18-fold increase in the number of mitochondrial genes for the smallest effect size (Table 3) to reach statistical significance. To provide biological context for the small differences in MK summary statistics that we observed between genomes, we calculated and contrasted effect sizes as the difference in means between genomes divided by the root mean square of the SD for *NI* and Z*. Across MK tables, the differences in *NI* between mitochondrial and autosomal genes yielded effect sizes that range from 0.18 to 0.71, smaller than those reported in the meta-analyses of Weinreich and Rand (2000), where the difference in mean *NI* between mitochondrial and nuclear genes was 3.2, with an effect size of 0.96. In an analysis of 98 nuclear loci in *D. melanogaster*, Presgraves (2005) reported significant differences in Z* for genes located in regions of high and low recombination for which the effect size was 0.96, over twice that which we observed between mitochondrial and autosomal gene Z* (Table 3).

### *NI* does not differ between mitochondrial and nuclear genes in humans

**Figure 4:**
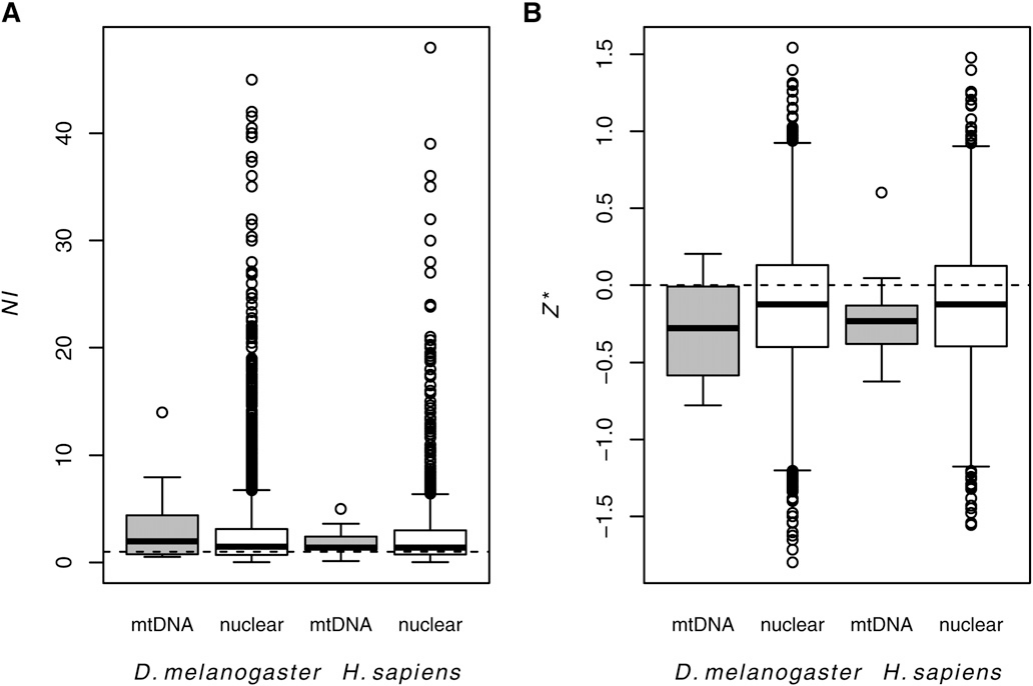
Distributions of (A) neutrality index (*NI*) and (B) Z* for mitochondrial and nuclear genes in *D. melanogaster* and *H. sapiens*. Three nuclear genes in flies and two nuclear genes in humans that had *NI* values greater than 50 were removed to improve visualization. Dashed lines represent the neutral expectation for each statistic. The *D. melanogaster* mitochondrial DNA (mtDNA) and nuclear sets contained 13 and 6113 genes, respectively. The *H. sapiens* mtDNA and nuclear sets contained 13 and 11,624 genes, respectively. See Table S2 and Table 3 and main text for statistical results.

Summary statistics of the MK table also did not differ between mitochondrial and nuclear genes in *H. sapiens* (*NI*, *P_MWU_* = 0.657; Z*, *P_MWU_* = 0.243), nor did the site-frequency spectrum differ between nonsynonymous and synonymous mitochondrial polymorphisms in humans (heterogeneity test, *P* > 0.36 for all genes). Values of *NI* and Z* for mitochondrial genes in humans were well within the distribution of these statistics for nuclear genes (Figure 4), and the confidence intervals around *NI_TG_* for the mitochondrial and nuclear genomes were overlapping (Table 3). Similar to the fly mtDNA and nuclear genome, the median values of *NI* and Z* for both the human mtDNA and nuclear genome were consistent with a slight excess of nonsynonymous polymorphism (Table 3). The distributions of *NI* and Z* were also largely overlapping and did not differ significantly between *D. melanogaster* and *H. sapiens* mitochondrial genes (*NI*, *P_MWU_* = 0.545; Z*, *P_MWU_* = 0.441) (Figure 4), despite differing nuclear *N_e_* between these species. This further supports the idea that the efficacy of purifying selection in these mitochondrial genomes is largely independent of *N_e_*.

Using data from flies and humans, we tested whether contrasts between nuclear and mitochondrial genes with similar function in OXPHOS and putatively similar selective effects of mutations (*s*) would reveal greater differences in *NI* between mitochondrial and nuclear genomes as a function of differing *N_e_*. For humans, there was no difference in *NI* or Z* between OXPHOS genes encoded in the mitochondrial and nuclear genomes (*P_MWU_* > 0.46 for both statistics), whereas for flies there was a weakly significant difference that was driven by the fact that the nuclear OXPHOS genes in our sample had values of *NI* and Z* that were more consistent with an excess of nonsynonymous substitutions (*NI*, *P_MWU_* = 0.026; Z*, *P_MWU_* = 0.022) (Figure S2). However, these data should be treated with some caution, as there were only 11 genes in our nuclear data set annotated to have OXPHOS function, and nine of these genes are part of Complex I (*ND*). Mitochondrial *ND* genes accumulate more amino acid substitutions than do other OXPHOS-complex genes in *Drosophila* (Ballard 2000; Montooth *et al.* 2009), potentially reflecting differences in functional constraint among complexes that are consistent with the OXPHOS-complex differences in *NI_TG_* that we observed in this study (Table 3 and Table S3).

Finally, we used the human data to illustrate the sensitivity of *NI* to sampling. When only a few individuals are sampled, the choice of genomes can lead to high variability and extreme values in *NI*, potentially as a result of single haplotypes that may carry multiple polymorphisms, as appears to be the case for human *ND6* (Table 4 and Table S5). For example, depending on which Japanese individuals we included in our analyses, *NI* for *ND6* takes on values of 30.71 (MK test, *P_FET_* = 0.001), 5.50 (*P_FET_* = 0.308), or 1.79 (*P_FET_* = 0.522) when sampling only three mtDNAs. As more mtDNAs are sampled, *NI* and Z* for each mitochondrial gene become more similar to the neutral expectation (Table 4 and Table S5). Overall, these analyses using *D. melanogaster* and *H. sapiens* mitochondrial genomes highlight the sensitivity of these MK statistics to the number of genomes sampled, the amount of divergence between species, and the low levels of polymorphism in these genes.

**Table 4:** The sensitivity of *NI* to sampling

Sample	1 African		1 African		1 African		19 African-American		30 African-American	
	1 European		1 European		1 European		20 European-American*^c^*		30 European-American*^c^*	
	1 Japanese*^a^*		1 Japanese*^b^*		1 Japanese*^b^*					
	*NI**^d^*	Z*	*NI*	Z*	*NI*	Z*	*NI*	Z*	*NI*	Z*
*ATPase*	2.27	−0.37	2.78	−0.44	2.84	−0.45	1.62	−0.22	1.65	−0.22
*COI*	**12.50**	**-1.08**	6.89	−0.88	**10.33**	**-1.03**	**3.61**	**-0.56**	**3.68**	**-0.56**
*COII*	3.33	−0.52	5.80	−0.82	3.28	−0.52	0.86	−0.13	0.64	−0.01
*COIII*	2.36	−0.53	1.38	−0.14	1.60	−0.39	1.38	−0.23	0.95	−0.08
*Cyt-b*	**3.78**	**-0.57**	3.64	−0.55	3.64	−0.55	2.29	−0.36	3.23*	**-0.50**
*ND1*	1.58	−0.28	2.51	−0.43	2.63	−0.45	1.49	−0.20	1.49	−0.19
*ND2*	**5.86**	**-0.76**	**5.86**	**-0.76**	**7.73**	**-0.86**	3.59	−0.55	**2.95**	**-0.46**
*ND3*	2.83	−0.52	6.80	−0.77	2.83	−0.52	1.33	−0.28	1.52	−0.25
*ND4*	0.93	−0.09	0.52	0.05	0.52	0.05	**0.13**	**0.60**	**0.11**	**0.70**
*ND4L*	2.20	−0.34	2.63	−0.42	2.63	−0.42	5.00	−0.62	4.00	−0.54
*ND5*	2.00	−0.32	2.38	−0.39	2.23	−0.37	1.20	−0.09	1.44	−0.16
*ND6*	30.71*	**-1.22**	5.50	−0.74	1.79	−0.25	1.30	−0.20	1.17	−0.16
All coding	2.88*	**-0.46**	2.50*	**-0.40**	2.41*	**-0.39**	**1.46**	**-0.17**	**1.55**	**-0.19**
*NI_TG_* (C.I.)*^e^*	**2.85** (1.83, 4.99)		**2.60** (1.65, 3.91)		**2.56** (1.59, 3.81)		1.48 (0.90, 2.24)		1.59 (0.93, 2.36)	

*NI*, neutrality index; *ATPase*, ATP synthase; *CO*, cytochrome *c* oxidase; *Cyt-b*, *Cytochrome B*; *ND*, NADH dehydrogenase; C.I., confidence interval; MK, McDonald-Kreitman.

a MK table counts from Nachman *et al.* (1996).

b MK table counts as mentioned previously, but substituting two different, randomly chosen Japanese samples.

c MK table counts from African-American and European-American sequences sampled from (Just *et al.* 2008) and (Rubino *et al.* 2012) with the chimpanzee mitochondrial reference genome as an outgroup (Horai *et al.* 1995).

d A count of 1 was added to each cell when calculating *NI* for any locus with a zero count in any cell. Values in bold indicate *P* ≤ 0.05; * indicates significant sample-wise Bonferroni-corrected *P*-value of less than 0.004 for Fisher’s exact test of the MK table.

e Calculated as in Table 3. No sample rejected Woolf’s test of homogeneity (*P* > 0.19 for all samples). Values in bold indicate that the confidence intervals do not overlap the neutral expectation of 1.

## Discussion

Using a large sample of whole-genome sequence data, we have tested a number of hypotheses about mtDNA evolution, and about differences in the efficacy of selection on mitochondrial *vs.* nuclear genes. Our data confirm that mtDNA do not have a signature of recombination and have lower silent-site diversity than do nuclear genes in *D. melanogaster*, which supports the prediction that the mitochondrial genome has a lower *N_e_* than the nuclear genome. We also show a skew in the site-frequency spectrum toward rare alleles in *D. melanogaster* that likely has two sources: 1) the accumulation of new mutations on what appears to be a mtDNA haplotype that has swept to high frequency in the recent past, and 2) the ancestral polymorphisms contained on migrant or remnant haplotypes that are now rare in this population. Despite the apparent reduction in *N_e_* for mtDNA, our findings indicate that selection is similarly effective at purging deleterious polymorphisms from the mitochondrial and nuclear genomes of *D. melanogaster*, and that the same is true in *H. sapiens*. Although all genomes that we analyzed showed some evidence of an excess of nonsynonymous polymorphism relative to the neutral expectation, the only significant differences in *NI* and Z* were between *D. melanogaster* mitochondrial genes and X-linked genes. X-linked genes in *Drosophila* have a greater proportion of beneficial substitutions than do autosomes (Langley *et al.* 2012; Mackay *et al.* 2012; Meisel and Connallon 2013; Garrigan *et al.* 2014), suggesting that what differs between mitochondrial genes and nuclear genes is likely the fraction of beneficial substitutions rather than the efficacy of purifying selection, which appears to be largely independent of *N_e_* in the *D. melanogaster* and *H. sapiens* mitochondrial genomes that we have analyzed.

Given its uniparental and haploid transmission, the expectation under neutrality is that the mtDNA has one-quarter the population size of the autosomes. This reduced value of *N* (and subsequently *N_e_*) matches that expected for the Y (or W) chromosome, and, like the Y chromosome, the mtDNA has little to no recombination. However, very much unlike the Y chromosomes that have been sequenced (*e.g.*, Charlesworth and Charlesworth 2000; Carvalho *et al.* 2009; Carvalho and Clark 2013; Bellott *et al.* 2014), animal mtDNA genomes do not show an accumulation of transposable elements, and the gene content of the animal mitochondrial genome is remarkably stable, with few gene losses and even fewer pseudogenes (Boore 1999; Ballard and Rand 2005). Furthermore, *d_N_*/*d_S_* is two to 15 times lower for mitochondrial genes than for nuclear genes in mammals (Popadin *et al.* 2013), and average values of *d_N_*/*d_S_* for mitochondrial genes are well under 0.1 and are, on average, only 13% that of nuclear genes in *Drosophila* (Bazin *et al.* 2006; Montooth *et al.* 2009). This pattern of amino acid conservation is particularly striking, given that the mutation rate in the *D. melanogaster* mtDNA is an order of magnitude greater than the per-site mutation rate in the nuclear genome, with an extreme bias toward nonsynonymous mutations in the mitochondrial genome (Haag-Liautard *et al.* 2007; Haag-Liautard *et al.* 2008). Although heteromorphic Y chromosomes do show signatures of less effective purifying selection, such as proliferation of satellite repeats and reduced codon bias (Bachtrog 2013; Singh *et al.* 2014), the single copy, X-degenerate genes that have remained on the human Y chromosome experience effective purifying selection (Rozen *et al.* 2009; Bellott *et al.* 2014), as do the protein sequences of *Drosophila* Y-linked genes (Singh *et al.* 2014). Thus, despite early loss of many genes when heteromorphic Y chromosomes and mtDNA formed, both these nonrecombining chromosomes contain genes maintained by effective purifying selection in the presence of reduced *N_e_*.

Many researchers have cited the early work on *NI* in *Drosophila* and mammals in support of the idea that mtDNA accumulate deleterious mutations (*e.g.*, Meiklejohn *et al.* 2007; Green *et al.* 2008; Neiman and Taylor 2009; Akashi *et al.* 2012). In fact, this idea has become so engrained that it is regularly cited in reviews of mitochondrial gene evolution (*e.g.*, Ballard and Whitlock 2004; Lynch 2007). What is perhaps surprising about this conversion of a small set of intriguing initial studies into dogma is that the early studies themselves were quite circumspect about the implications of their results. For instance, Nachman (1998), in noting that very few nuclear loci were available for comparison, stated “It is also unclear whether the patterns reported here are unique to mitochondrial DNA.” Data from the few nuclear genes that had been sequenced raised “the possibility that the patterns reported here for mtDNA may also be found at some nuclear loci” (Nachman 1998). Even studies that did have access to additional nuclear datasets were only able to calculate *NI* for 36 nuclear loci (Weinreich and Rand 2000), and the *NI* values that were available often did not deviate significantly from neutrality (Nachman 1998; Weinreich and Rand 2000). Those that did reject neutrality tended to do so weakly, perhaps due to the small number of polymorphisms in mitochondrial samples even when the number of individuals sampled is high (*e.g.*, *ND3*, Nachman *et al.* 1996). Nevertheless, there are mitochondrial genes that do strongly reject neutrality, and some of these had *NI* values that greatly exceeded *NI* for the sampled nuclear loci. On the basis of these and similar comparisons, many authors have reached the conclusion that mtDNA evolves in a manner distinct from the nuclear genome. Our results using the whole genomes of flies and humans, combined with observations of low mitochondrial *d_N_*/*d_S_*, suggest that the mitochondrial genomes of flies and humans are not suffering less effective purifying selection relative to the nuclear genome, and that differences in selection between these genomes may lie in differing rates of adaptive evolution.

Reductions in *N_e_*—due either to reductions in census population size or to the increased effect of linked, selected variants in regions of low recombination—are expected to result in a reduction in the efficacy of purifying selection. Indeed, comparisons of MK test results across a range of species with different values of *N_e_* have revealed this expected relationship (*e.g.*, Li *et al.* 2008; Wright and Andolfatto 2008; Gossmann *et al.* 2010), as have comparisons of *NI* across regions of the *D. melanogaster* nuclear genome with different recombination rates (Presgraves 2005; Langley *et al.* 2012). Therefore, all things being equal, mitochondrial loci would be expected to harbor an excess of nonsynonymous polymorphisms relative to nuclear loci due to reduced *N_e_*. Our results suggest that all things are not equal between these two cellular compartments, and that there may be features of the mitochondrion that make it less likely to accumulate deleterious mutations. One such feature is the “bottleneck” that occurs in the number of mtDNAs that are passed from mother to offspring—this event makes it possible for selection to act within hosts, possibly increasing the power of selection to remove deleterious mutations (Bergstrom and Pritchard 1998; Rand 2011) and reducing variability in mitochondrial *N_e_* among taxa, relative to nuclear genomes. The additional layers of selection imposed by mitochondrial inheritance, combined with stronger negative selective effects of amino acid changing mutations in mitochondrial genes (*e.g.*, Popadin *et al.* 2013), may allow the mtDNA to escape the accumulation of deleterious mutation, resulting in relatively similar values of *NI* between nucleus and mitochondria. If the selective effects of mutations in mitochondrial genes are beyond the “horizon” where all mutations will behave similarly regardless of *N_e_* (Nachman 1998; Eyre-Walker and Keightley 2007), then we expect patterns of mitochondrial polymorphism and divergence to be largely independent of *N_e_*.

Our results come with several caveats. First, we have only studied two organisms—it may be that a more comprehensive review of *NI* in mtDNA and nuclear loci across many species will reveal a difference in the average efficacy of purifying selection or highlight lineage-specific patterns. The early meta-analyses of *NI* contained loci from a wide range of animals (Nachman 1998; Weinreich and Rand 2000), and using data from only *Drosophila* and humans may provide a limited perspective. Nevertheless, these are two model organisms for evolutionary biology that span a large range of mtDNA:nuclear substitution rates, and studies of these species have led the way for much of modern population genetics. Second, it is clear from our analysis of the *D. melanogaster* mtDNA that it is not at equilibrium, and may be recovering from a partial cytoplasmic sweep that may be associated with *Wolbachia* (Richardson *et al.* 2012). Much of the theory used to predict *NI* values from *N_e_* and *s* assumes mutation-selection-drift balance (see, *e.g.*, Nachman 1998), and deviations from this equilibrium can result in more complex relationships between *N_e_*, *s*, and *NI* (Messer and Petrov 2013). Although nonequilibrium histories may mean that mtDNA *NI* values are not at equilibrium, it is equally likely that nuclear genes from *D. melanogaster* are not at mutation-selection-drift equilibrium (Hahn 2008; Langley *et al.* 2012). Whether or not the mtDNA is at equilibrium, and whether or not the *NI* values calculated from this snapshot of two species represent equilibrium values, our results still imply that there is little difference between nuclear and mitochondrial measures of the efficacy of purifying selection.

Despite the mitochondrial genome experiencing a distinct population genetic environment relative to the nuclear genome, our whole-genome analyses uncovered little evidence for an excess accumulation of slightly deleterious mutations in mitochondrial genomes, relative to nuclear genomes. In fact, the only strong evidence for a reduced efficacy of selection in animal mtDNA, relative to nuclear genomes, comes from comparative studies of nuclear and mitochondrial tRNAs (Lynch 1996; Lynch 1997). As discussed previously in this article, in the absence of a pattern in *NI*, there are few patterns of molecular evolution in animal mtDNA indicative of deleterious mutation accumulation [but see Osada and Akashi (2012)]. This pattern is in stark contrast to the patterns found in analogous nuclear regions with reduced *N_e_* and low recombination, like the Y chromosome. Determining whether mtDNA accumulate deleterious polymorphisms and substitutions more readily than nuclear DNA in a larger sample of species (and what type of loci may be affected) will be a particularly fruitful avenue for future studies.

## 
